# National trends in lumbar spine decompression and fusion surgery in Finland, 1997–2018

**DOI:** 10.1080/17453674.2020.1839244

**Published:** 2020-10-27

**Authors:** Ville T Ponkilainen, Tuomas T Huttunen, Marko H Neva, Liisa Pekkanen, Jussi P Repo, Ville M Mattila

**Affiliations:** a Department of Orthopaedics and Traumatology, Tampere University Hospital, Tampere;; b Department of Anesthesiology, Tampere University Hospital, Tampere;; c Department of Surgery, Central Finland Hospital District, Jyväskylä;; d Faculty of Medicine and Health Technology, Tampere University, Tampere;; e COXA Hospital for Joint Replacement, Tampere, Finland

## Abstract

Background and purpose — During recent years, spine surgery techniques have advanced, the population has become older, and multiple high-quality randomized controlled trials that support surgical treatment for degenerative spinal stenosis and spondylolisthesis have been published. We assess the incidence and trends in spine fusion and decompression surgery in Finland between 1997 and 2018.

Patients and methods — We used nationwide data from the Finnish nationwide National Hospital Discharge Register. The study population covered all patients aged 20 years or over in Finland (5.5 million inhabitants) during a 22-year period from 1997 through 2018. All patients who underwent spinal decompression were included. Patients with both decompression and fusion codes were analyzed as fusions.

Results — 76,673 lumbar spine decompressions and fusions were performed during the study period. The incidence of lumbar spine decompressions increased from 33 (95% CI 23–45) per 100,000 person-years in 1997 to 77 (CI 61–95) per 100,000 person-years in 2018. The incidence of lumbar spine fusions increased from 9 (CI 5–17) per 100,000 person-years in 1997 to 30 (CI 21–43) per 100,000 person-years in 2018. The increase in incidence of lumbar spinal fusions was highest among women aged over 75 years, with a 4-fold increase.

Interpretation — The incidence of lumbar spine fusions and decompressions increased between 1997 and 2018 in Finland. These findings may be the result of the emergence of advanced surgical techniques but may also be the result of an aging population and increased evidence supporting the surgical treatment of various spinal pathologies.

During recent years, there has been an increasing trend for the surgical treatment of degenerative spine pathologies (Gray et al. 2006). For example, more than 240 000 decompression procedures were performed in the United States in 2007. In 2004, the total cost of spine fusion and decompression surgeries in the United States was estimated to be 21 billion USD (Deyo 2007). Previous studies from the US have shown that the incidence of surgically treated degenerative spine pathologies slowly increased between 1980 and 1990 (Taylor et al. 1994). Thereafter, the rates rapidly increased from 1990 to 2010 (Deyo et al. 2005, Patil et al. 2005, Passias et al. 2017). According to a study by Seitsalo (1996), the incidence of lumbar spine fusion operations in Finland increased by 103% between 1987 and 1994. Conversely, they also reported that the incidence of lumbar spine decompression decreased by 12% during the same period. Recent trends in lumbar spine fusion and decompression surgery in Finland are not known, however.

Spine surgery has undergone many evolutionary steps during the previous decades (Patil et al. 2005, Kim et al. 2011). Of these, new, less invasive techniques (Kim et al. 2011), better availability, higher quality of MRI (Patil et al. 2005), and a better understanding of degenerative spine pathologies (Patil et al. 2005, Passias et al. 2017) have all been presented as major factors behind the increasing incidence of spine surgery. Most importantly, multiple high-quality randomized controlled trials (RCT) supporting the operative treatment of degenerative spinal stenosis (Weinstein et al. 2008, 2010, Lurie et al. 2015) and spondylolisthesis (Weinstein et al. 2007, Ekman et al. 2009, Weinstein et al. 2009) have recently been published. By contrast, the effect of spinal fusion is debated (Försth et al. 2016).

Taking into account the increasing trends in degenerative spine surgery and recent scientific evidence supporting surgery, we evaluated the changes in the incidence of spinal fusion and decompression surgery in Finland between 1997 and 2018. 

## Patients and methods

The data for this study was obtained from the Finnish National Hospital Discharge Register (NHDR). Patient characteristics, such as age, sex, diagnosis, and operations performed during the hospital stay, were obtained from the register. We included all patients aged 20 years or over. In Finland, it is mandatory for all public and private Finnish hospitals to collect data for the NHDR, and, as a result, the procedural coding has coverage and accuracy of more than 90% (Mattila et al. 2008, Sund 2012). However, the limitations of the NHDR are that it does not include the laterality of the surgery, comorbidities, or many clinically relevant risk factors, such as smoking or the use of alcohol (Sund 2012).

The main outcome for this study was the number and incidence of spine surgeries in the NHDR performed with pre-defined procedures. We included hospitalizations with the following NOMESCO (Nordic Medico-Statistical Committee, Finnish version approved by the Finnish Institute for Health and Welfare) classification procedure codes: ABC36 (Decompression of lumbar nerve roots), ABC56 (Decompression of lumbar spinal canal and nerve roots), ABC66 (Decompression of lumbar spinal channel), NAG60 (Anterior fusion of lumbar spine with fixation), NAG61 (Posterior fusion of lumbar spine without fixation), NAG62 (Posterior fusion of lumbar spine with fixation, 2–3 vertebrae), NAG63 (Posterior fusion of lumbar spine with fixation, more than 3 vertebrae), NAG65 (Anterior and posterior fusion of lumbar spine), NAG66 (Posterior interbody fusion of lumbar spine, 2 vertebrae), and NAG67 (Posterior interbody fusion of lumbar spine, more than 2 vertebrae). The ABC codes listed above were defined as lumbar decompression surgery and the NAG60–67 codes were defined as lumbar fusion surgery. Patients with both decompression and fusion codes were analyzed as fusions. For those patients with the aforementioned lumbar spine surgeries, the International Classification of Diseases, Tenth Revision (ICD-10) diagnosis codes were also evaluated, and patients with M00-M99 (Diseases of the musculoskeletal system and connective tissue) and G00-G99 (Diseases of the nervous system) codes were included. Patients with spine fracture (S1*, S2*, and S3*), sarcoma (C40–41 and C45–C49), and metastases (C76–79) were excluded.

If a patient underwent multiple operations with the aforementioned diagnosis and operation codes during the follow-up, the first operation was considered to be the primary operation, and the time between the first and the second operations was calculated. All operations performed under the operation and diagnosis codes described above were considered to be secondary operations. The patients were categorized into 3 age groups: between 20 and 54 years, between 55 and 74 years, and 75 years and older.

### Statistics

The incidence rates were calculated using the annual mid-populations obtained from the national population register (Statistics Finland). The annual incidence rates (per 100,000 person-years) were based on the entire adult population of Finland and stratified according to sex and age. As the Finnish NHDR does not include death or other competing risks, we did not perform further survival analysis. 95% confidence intervals (CI) were calculated for incidence rates by using the Poisson exact method. Calculations were performed using R version 3.6.2 (R Foundation for Statistical Computing, Vienna, Austria).

### Ethics, funding, and potential conflicts of interest

This study was performed as a retrospective register study, and therefore ethical approval was not required. The study received approval from the Finnish Institute for Health and Welfare Dnro: THL/1800/5.05.00/2019. This study has not received any external financial support. None of the authors have any conflicts of interests to declare.  

## Results

**Figure 1.**
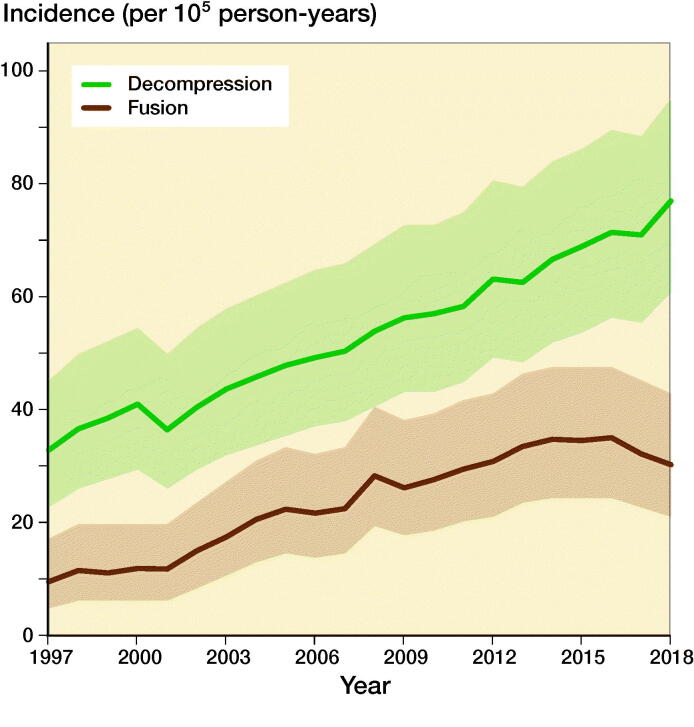
Incidence of lumbar spine decompression and fusion operations in Finland between 1997 and 2018.

76,673 lumbar spine decompressions (69%) and fusions (31%) were performed in Finland between 1997 and 2018 according to the Finnish NHDR. The mean (SD) age of all patients was 63 (13) years. Females accounted for 55% (n = 42,454) of the operated patients. The annual population-based incidence of all lumbar decompressions and spine fusions increased by 155%, from 42 per 100,000 person-years in 1997 to 107 per 100,000 person-years in 2018 (Figure 1). From now on incidence refers to number per 100,000 person-years.

The incidence of lumbar spine decompressions increased by 133%, from 33 (CI 23–45) in 1997 to 77 (CI 61–95) in 2018 (Figure 1). The proportions of fusion and decompression operations remained similar throughout the study period, with decompressions accounting for 78% in 1997 and 72% in 2018. The mean (SD) age of the patients who underwent spinal decompression surgery was 64 (13) years, and the mean age for patients who underwent spinal fusion was 59 (14) years. The changes in the incidence of decompression surgery were similar among both males and females (Figure 2).

Figure 2.Incidence of lumbar spine decompression and fusion operations by gender and age group in Finland between 1997 and 2018.

**Figure:**
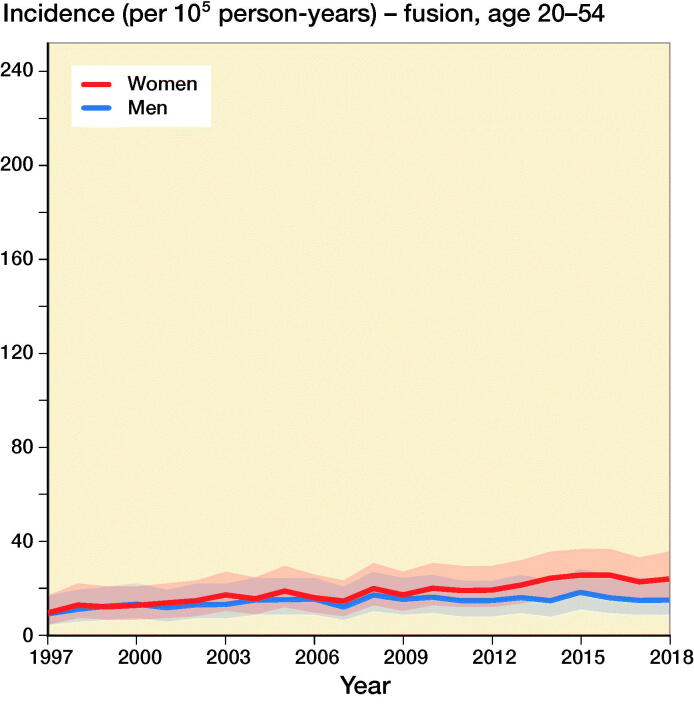

**Figure:**
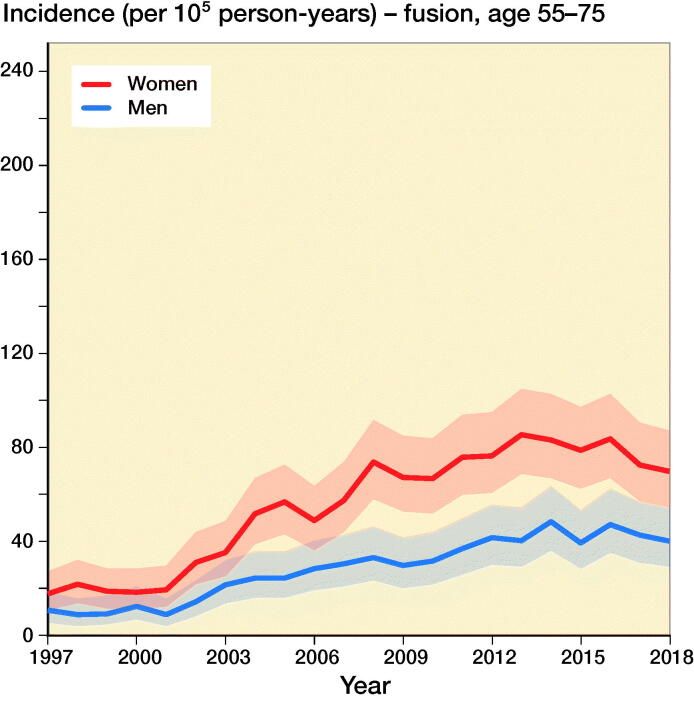

**Figure:**
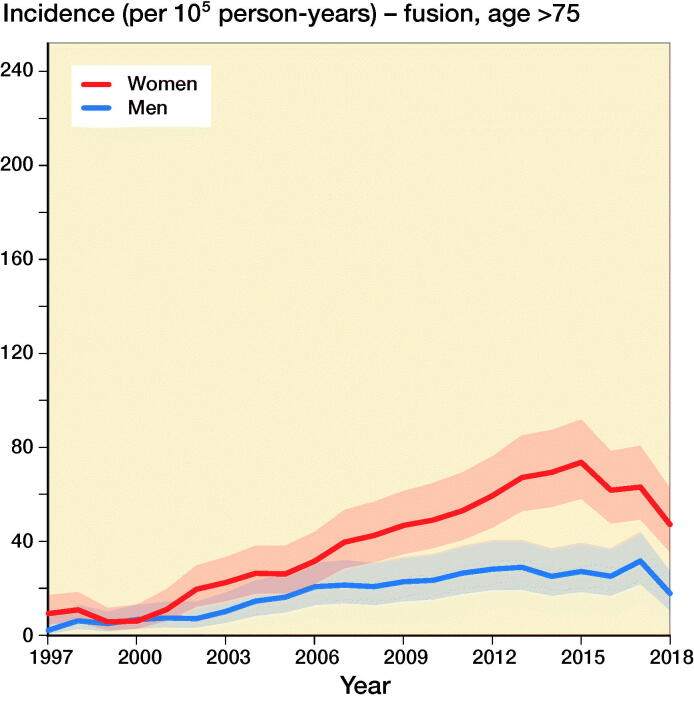

**Figure:**
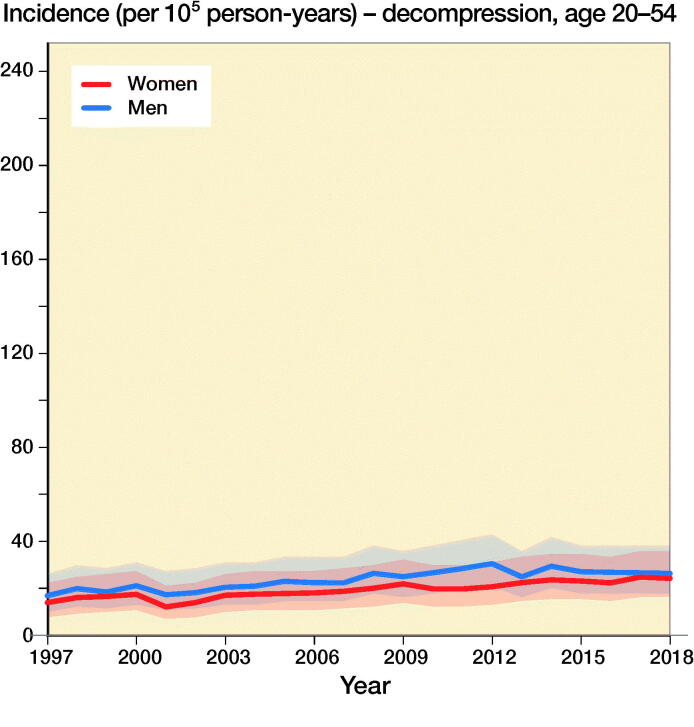

**Figure:**
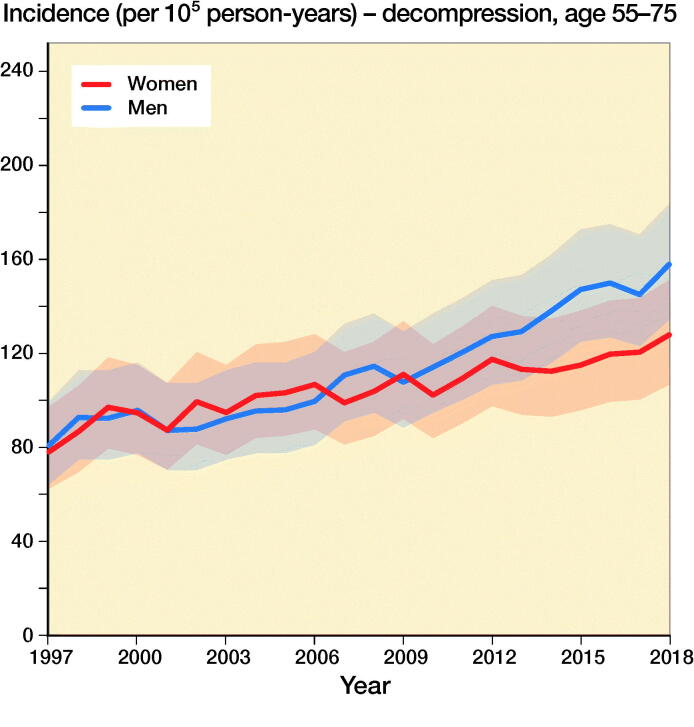

**Figure:**
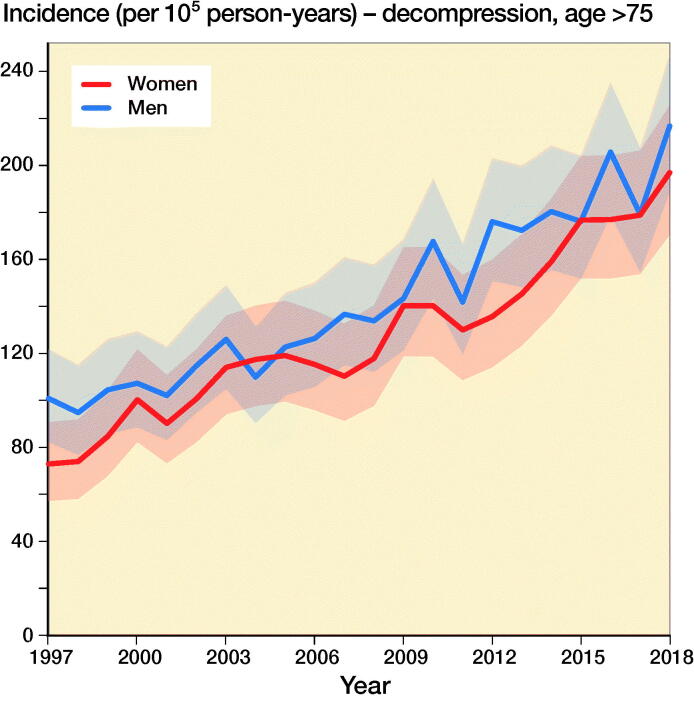

The incidence of lumbar spine fusions increased by 289%, from 9 (CI 5–17) in 1997 to 35 (CI 24–48) in 2016 (Figure 1). After 2016, the incidence decreased and was 30 (CI 21–43) in 2018. The incidence of lumbar spine fusion surgery rose fastest among women aged over 75 years. In this group, incidence increased from 9 (CI 5–17) to 47 (CI 35–63) (Figure 2), an increase of 422%. The change was also notable among women aged between 55 and 74 years, with a 289% increase in incidence from 18 (CI 11–27) to 70 (CI 55–87) between 1997 and 2018. The incidence of lumbar spine fusion in women aged under 55 years remained low. However, incidence still increased by 140% in this group, from 10 (CI 5–17) in 1997 to 24 (CI 16–36) in 2018.

**Figure 3.**
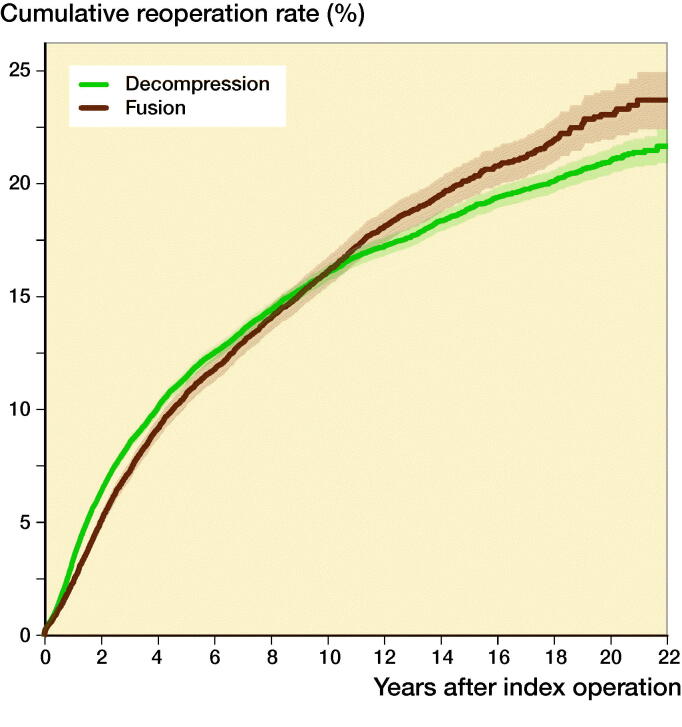
Cumulative reoperation rate of lumbar spine decompression and fusion operations in Finland between 1997 and 2018.

The cumulative reoperation rate of decompression and spinal fusion was comparable for the first 10 years of follow-up (Figure 3). Thereafter, the reoperation rate of spinal fusion started to rise slightly faster than that of decompression, achieving a reoperation rate of 23%, whereas decompression achieved a reoperation rate of 21% after 22 years of follow-up.  

## Discussion

Our main finding was that the nationwide population-based incidence of lumbar spine decompressions and fusions in Finland increased by 155% between 1997 and 2018. Lumbar spine fusion surgery increased by 289% until 2016, and then decreased slightly. Conversely, spinal decompression operations increased by 133% from 1997 to 2018. The most notable change in spinal fusion operations was among women over 75 years of age, as the incidence increased by 422% during our study period. The change was also notable among women aged between 55 and 74 years. The incidence in this group increased by 289%.

This increasing trend in lumbar fusion surgery is in line with the findings of previous studies. An increase in lumbar spinal fusion surgery was reported in the United States in the 1990s, as the incidence doubled between 1979 and 1990 (Taylor et al. 1994). After 1990, the increase in lumbar spine fusions grew steeper, with a 220% increase in incidence between 1990 and 2001, reaching an incidence of 61 per 100,000 person-years (Deyo et al. 2005). The slope became even steeper after 1996 when the US Food and Drug Administration approved the use of intervertebral cages, a new type of spinal implant. In Finland, Seitsalo (1996) reported that the incidence of lumbar spine fusion operations increased by 103%, from 3.7 per 100,000 person-years in 1987 to 7.5 per 100,000 person-years in 1994. Moreover, Seitsalo also reported that the incidence of lumbar spine decompression (excluding lumbar discectomies) was 33 per 100,000 person-years in 1987 and 29 per 100,000 person-years in 1994, a decrease of 12%.

During the late 1990s, the incidence of spinal fusion surgery in our data increased rapidly (289% vs. 103%), although the trend decreased slightly after 2016. The incidence of spinal decompression also began to rapidly increase during the same time period (133% vs. 12%). Patil et al. (2005) have suggested that advancements in diagnostics and the availability of MRI may be behind the increases in the incidence of cervical spinal operations in the United States (Patil et al. 2005). Due to increased knowledge, these pathologies have become more familiar to a larger group of physicians, and patients will therefore be more likely to be referred to a neurosurgeon or orthopedic spine surgeon.

Additionally, the increase in both decompressions and fusions was most notable among patients aged 75 years and older. This change is probably partly explained by people living longer and the fact that older individuals in general are in better health and are more active than previous generations. The demands of older individuals might therefore be higher. Thus, there are more patients aged over 75 years who can benefit from surgery. Furthermore, the threshold for surgery may have also been lowered due to changes in surgical techniques and advancements in the perioperative care of the elderly. Along with the general advancements in medicine and surgery, more accessible resources in preoperative examinations, improvements in preoperative and postoperative care protocols, and enhancements in surgical practice, it is now much easier to offer operative treatment to older patients. As a result of the evolution of surgical practices, operation times have decreased markedly. Therefore, it is possible that the overall perioperative efficiency has increased the availability and thus the incidence of surgical treatment in Finland. Moreover, it is evident that there is more good quality scientific evidence supporting surgical treatment. Multiple RCTs were published between 2007 and 2015 that supported operative treatment for spinal stenosis (Malmivaara et al. 2007, Weinstein et al. 2008, 2010, Slätis et al. 2011, Lurie et al. 2015). 2 of the studies were conducted by a Finnish research group (Malmivaara et al. 2007, Slätis et al. 2011). These studies might have also affected the increasing trends in lumbar spine surgery.

The incidence of spinal fusion surgery decreased after 2016, whereas the increasing trend in spinal decompression surgery continued. A possible explanation for this might be a Swedish RCT by Försth et al. that was published in 2016. The study compared decompression plus fusion surgery and decompression surgery alone in the treatment of spinal stenosis. Their most important finding was that decompression combined with fusion did not result in better pain relief or functional outcomes at 2 or 5 years when compared with decompression alone The strengths of their study were the high number of patients (n = 247) and long follow-up time (5 years) combined with a high-quality study setting. Thus, the conclusions of the study might have affected the decrease in spinal fusion surgery and the increase in spinal decompression surgery in Finland after 2016. Another explanation for the decrease in spinal fusion surgery is the low incidence of reoperations. In degenerative diseases, the degeneration of adjacent levels continues after the primary surgery and may result in adjacent level disease and reoperation. We analyzed only re-decompressions and re-fusions. Notably, the cumulative reoperation rates of spinal fusion and decompression were comparable for the first 10 years. After that, however, the reoperation rate of spinal fusion started to rise slightly faster than that of decompression, achieving a reoperation rate of 23%, whereas spinal decompression achieved a reoperation rate of 21% after 22 years of follow-up. These nationwide rates are notably lower than in the previous study by Irmola et al. (2018), partly due to our only including re-decompressions and re-fusions.

The incidence of spinal decompression combined with fusion was higher among women than men. It has previously been noted that spinal stenosis is slightly more common among women than men in northern countries (Lønne et al. 2019). One of the reasons for this difference is that women have lower density of the paraspinal muscles, which more often leads to low back pain and spinal degeneration (Bulcke et al. 1979, Kalichman et al. 2010) The density of the paraspinal muscles is also known to decrease with higher age (Kalichman et al. 2010). Both factors may have played a role in the observed increased incidence of operative treatment of symptomatic degenerative spinal pathologies among women (especially older women).

A clear limitation of our study is that we were unable to identify whether a reoperation was performed on the same site in the spine. Additionally, due to the retrospective register nature of the study, we were unable to reliably assess the reason for reoperation. These limitations can also affect our reoperation rates because part of the operations categorized as reoperations might have been operations on another site. The strength of our study is the high-quality nationwide register data collected from the NHDR database. The database includes all operations performed in all private and public hospitals, and therefore the quality of the data has been shown to be excellent in terms of coverage and accuracy (Keskimaki 1991, Mattila et al. 2008, Sund 2012). In addition to the coverage, the strength of the data is that each individual patient can be followed by their personal ID numbers, and we were therefore able to observe all operations these patients underwent during the study period.

## Conclusions

The annual population-based incidence of lumbar spine decompressions and fusions increased by 155% between 1997 and 2018. Moreover, lumbar spine fusion surgery increased rapidly until 2016, and then started to decrease slightly. Conversely, the number of spinal decompression operations increased continuously between 1997 and 2018. The most notable change in spinal fusion operations was among women aged 75 years and older, as the incidence increased by 422% during our study period. The reasons behind the changes may be the aging population, advances in diagnostics and surgical techniques, and increased evidence supporting the surgical treatment of various lumbar spine pathologies.
